# Supplementary material for: Stacking Model‐Based Classifiers for Dealing With Multiple Sets of Noisy Labels
Source: Biom J. 2025 Mar 12;67(2):e70042. doi: 10.1002/bimj.70042 (PMC11898607; doi:10.1002/bimj.70042)
Supplement: Supplementary file 1 — Supporting Information [file BIMJ-67-e70042-s001.zip › density-based-ensemble-model-main/competitors_real_data/netlab-master/netlab-master/help/demmdn1.htm]

Netlab Reference Manual demmdn1


# demmdn1

## Purpose

Demonstrate fitting a multi-valued function using a Mixture Density Network.

## Synopsis

```
demmdn1
```

## Description

The problem consists of one input variable
`x` and one target variable `t` with data generated by
sampling `t` at equal intervals and then generating target data by
computing `t + 0.3*sin(2*pi*t)` and adding Gaussian noise. A
Mixture Density Network with 3 centres in the mixture model is trained
by minimizing a negative log likelihood error function using the scaled
conjugate gradient optimizer.

The conditional means, mixing coefficients and variances are plotted
as a function of `x`, and a contour plot of the full conditional
density is also generated.

## See Also

`mdn`, `mdnerr`, `mdngrad`, `scg`

---

**Pages:**
Index


---

Copyright (c) Ian T Nabney (1996-9)
